# Periodontal Therapy and Systemic Inflammation in Type 2 Diabetes Mellitus: A Meta-Analysis

**DOI:** 10.1371/journal.pone.0128344

**Published:** 2015-05-26

**Authors:** Hilana Paula Carillo Artese, Adriana Moura Foz, Mariana de Sousa Rabelo, Giovane Hisse Gomes, Marco Orlandi, Jean Suvan, Francesco D’Aiuto, Giuseppe Alexandre Romito

**Affiliations:** 1 Division of Periodontics, Department of Stomatology, School of Dentistry, University of São Paulo, São Paulo, SP, Brazil; 2 Unit of Periodontology, UCL Eastman Dental Institute, London, United Kingdom; Canadian Agency for Drugs and Technologies in Health, CANADA

## Abstract

**Aim:**

The aim of this systematic review was to assess the effect of periodontal therapy (PT) on serum levels of inflammatory markers in people with type 2 diabetes mellitus (T2DM).

**Methods of Study Selection:**

A literature search was carried out using MEDLINE via Pubmed, EMBASE, LILACS and Cochrane Central Register of Controlled Trials (CENTRAL) databases. Randomized-controlled trials (RCTs) and controlled clinical trials (CCTs) evaluating the effect of PT on systemic inflammatory markers were deemed eligible. Case series (CS), reports and pilot trials were excluded. Study quality was assessed using the Cochrane Collaboration’s risk assessment tool. Meta-analysis was carried out using random effect methods.

**Results:**

The search strategy identified 3,164 potential studies of which 61 were assessed for eligibility and 9 (6 RCTs and 3 CCTs) were included in this systematic review. Three RCTs were classified by the authors as being at low risk of bias and three were “unclear” and classified as uncertain risk of bias. All CCTs were considered to be at a high risk of bias. The meta-analysis showed a statistically significant mean difference (MD) for TNF- α (-1.33 pg/ml, 95% CI: -2.10; -0.56, p<0.001) and CRP (-1.28 mg/l, 95% CI: -2.07; - 0.48, p<0.001) favoring periodontal intervention versus control.

**Conclusion:**

The results of this meta-analysis support the hypothesis that PT reduces serum levels of TNF- α and CRP in T2DM individuals. The decrease of inflammatory burden has important implications for metabolic control and can, in part, explain the mechanisms linking periodontitis and increased risk for complications in people with T2DM.

## Introduction

Periodontitis is a chronic inflammatory/infectious disease, characterized by the loss of both soft and hard tooth supporting tissues [1]. Dental biofilm is the main etiological factor for oral production of inflammatory biomarkers [2, 3], although several biological mechanisms may be involved. Local production of cytokines in response to periodontal bacteria and their products is related to higher serum concentrations of proinflammatory biomarkers [4]. Periodontal treatment (PT) is associated with a reduction in inflammatory burden in patients with periodontitis [5, 6]. Type 2 diabetes mellitus (T2DM) is a disorder with both metabolic and vascular components. It is characterized by hyperglycemia due to defective insulin function or altered insulin cell receptors (insulin resistance) rather than its deficiency [7]. Many factors contribute to the onset and development of diabetes complications, such as genetics, diet, lifestyle, age and obesity [8, 9]. Studies indicate that control of inflammatory processes may be related to novel approaches in treating this disorder [10–12]. T2DM is a recognized risk factor for periodontal diseases (PD) [13, 14], affecting their prevalence, incidence and severity [15]. Nevertheless, the relationship between both disorders is bidirectional [14–17]. Altered systemic inflammatory response has been recognized in both PD and T2DM [18]. PD may therefore represent an additional factor contributing to the total inflammatory burden in people with T2DM [19–21]. Recent systematic reviews reported beneficial effects of PT on the glycemic control in individuals with T2DM [22–24], although the largest clinical trial published to date was inconclusive [25], having limited focus on other systemic outcomes (i.e., inflammatory serum markers). The aim of this systematic review (SR) was to answer the following question: can PT affect serum inflammatory biomarkers in people with PD and T2DM?

## Materials and Methods

The protocol of this review was registered at the National Institute for Health Research PROSPERO, International Prospective Register of Systematic Reviews (http://www.crd.york.ac.uk/Prospero, registration 4 number CRD42012002988). We designed the protocol in accordance to Cochrane standards for analysis and reporting of methods. The search criteria met the Preferred Reporting Items for Systematic Reviews and Meta-analysis (PRISMA) guidelines [26].

### Criteria for considering studies for this review

#### Type of studies

Randomized controlled trials (RCTs) were considered the most appropriate study design to answer the research question for this review. Owing to the limited number of RCTs found, controlled clinical trials (CCTs) were also included.

#### Type of participants and inclusion/exclusion criteria

Participants were included if they were diagnosed with type 2 diabetes (according to the WHO criteria for diagnosis: fasting plasma glucose ≥ 126 mg/dl and/or 2h post-glucose challenge of 220 mg/dl). Further inclusion criteria were the following: (1) Participants having received PT with at least 3 months of follow-up; (2) assessment of serum inflammatory biomarkers related to insulin resistance; and (3) at least 30 individuals included in the type 2 diabetes group. All other study designs, such as case series reports and pilot studies, were excluded from this review. Trials in which inflammatory markers were not available for analysis (when original values could not be retrieved after contacting the original authors) were not eligible for inclusion.

#### Type of periodontal interventions

The periodontal interventions were based on professional oral hygiene instructions; full-mouth scaling and root planing (supra/subgingival biofilm and calculus removal) (SRP); surgical procedures (i.e., periodontal flap surgery) and SRP plus local or systemic antimicrobial. Periodontal interventions were compared with the passive option (no periodontal treatment).

### Types of outcome measures

#### Primary outcome

Serum laboratory markers of inflammation following PT.

#### Secondary outcome

Occurrence of adverse effects related to PT.

### Search Strategy

Identification of studies for this SR was performed through detailed search strategies developed for each database searched (MEDLINE, EMBASE, LILACS and Cochrane central register of controlled trials). Articles in the English language were included by searching MEDLINE via Pubmed (1950 to November 2013), the Cochrane Central Register of controlled trials (CENTRAL), EMBASE via Ovid (1980 to November 2013), and LILACS (1982 to November 2013). Reference lists of previous reviews and identified studies were manually examined (hand search) in an attempt to identify any additional articles. The databases Clinical Trials Gov and OpenGray were also searched to identify unpublished trials. Authors of the included manuscripts were contacted for missing data as needed.

### Study eligibility and data collection

All titles and abstracts (if available) were screened by 3 independent reviewers (M.S.R., A.M.F. and G.H.G.). Irrelevant records were excluded and full texts of potentially relevant studies were examined. Relevant data was extracted and recorded in duplicate (by M.S.R., A.M.F. and G.H.G.) using specially designed data-extraction forms: (1) citation, publication status and publication year; (2) center where the trial was performed; (3) study design; (4) participant characteristics; (4) outcome measures; and (6) conclusions. Discrepancies between extractors were resolved by discussion or with the help of a fourth reviewer (H.P.C.A.) who acted as an arbiter to resolve any disagreement.

### Quality of evidence

Quality assessment of selected studies was performed with the Cochrane Collaboration tool for assessing risk of bias [27].

### Diagnostic assessment

Adequate diagnosis of type 2 diabetes was defined when recorded by medical staff using the WHO diagnostic criteria 2006 [28] defined as fasting plasma glucose ≥ 126 mg/dl and/or 2h post-glucose challenge of 220 mg/dl. Diagnosis was deemed inadequate if made through other methods, and unclear when the methods used were not clear or not reported in the studies. Due to the heterogeneity of criteria and case definitions of periodontitis, we accepted any periodontitis diagnosis as defined by the authors.

### Data synthesis

Pooling of data was based on study design, outcomes, population characteristics, inflammatory mediators affected by periodontal treatment and types of PT. Specific inflammatory mediators that improved after therapy were also recorded. Descriptive summaries of included studies were entered into evidence tables and a narrative synthesis of evidence was performed. If outcome data could not be extracted from an article (e.g., data presented in a graph, use of median values) the corresponding author was asked to provide the data.

For meta-analysis, the mean difference (MD) and 95% confidence interval (CI) values among PT group and no-treatment group (control group) at both baseline and end were only calculated for two serum biomarkers: tumor necrosis factor alpha (TNF-α) and high sensitive C-reactive protein (hsCRP). Reviewers focused on these two markers based on the studies included in this review. Pooled estimates of the mean differences were calculated using random effects models in order to take potential inter-study heterogeneity into account and to adopt a more conservative approach. The statistical analysis was performed using the commercial statistical software Stata (Stata for Windows, v.11, Stata Corporation College Station, Texas, USA). The significant level established for analysis was 5% (p<0.05). The study by Chen et al. (2012) [29] had two treatment arms, and data from the control group was used in more than one comparison. Thus, the number of cases in this group was divided by number of comparisons (Chen et al., 2012a, Chen et al., 2012b) [29].

## Results

### Search results

**Fig 1:**
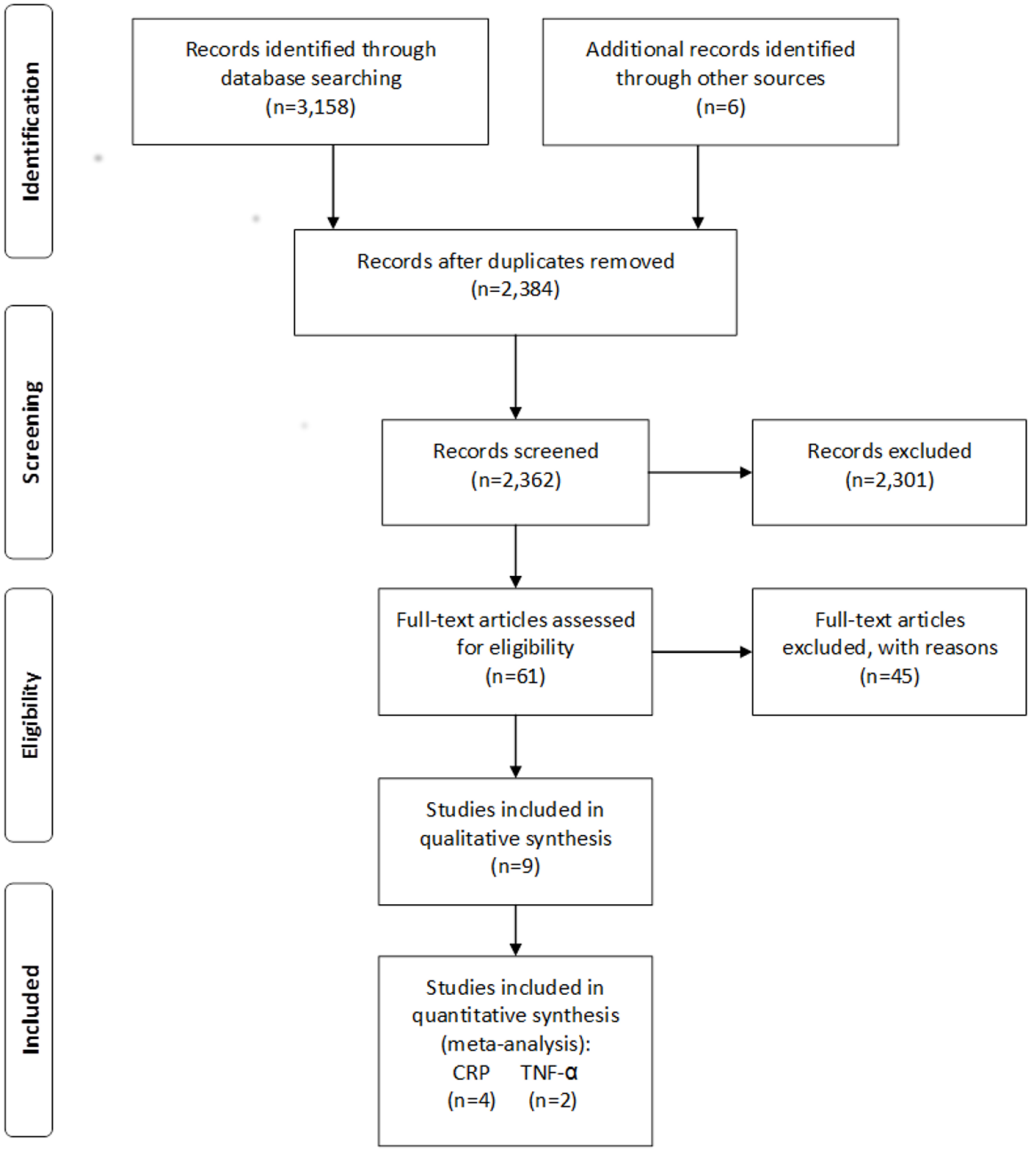
PRISMA 2009 Flow Diagram. From: Moher D, Liberati A, Tetzlaff J, Altman DG, The PRISMA Group (2009). Preferred Reporting Items for Systematic Reviews and Meta-Analysis: The PRISMA Statement. PLoS Med 6(6): e1000097. doi:10.1371/journal.pmed1000097. For more information, visit www.prisma-statement.org.

The initial search identified 3,164 potentially relevant articles, of which 2,301 were excluded based on their titles and abstracts. A second level, full-text search was performed on the 61 remaining studies. Nine studies were selected for full-text assessment with a total of 623 participants [5, 29, 30–36]. Of these, 4 studies were included in CRP meta-analysis [31, 33, 29, 35] and 2 in TNF-α meta-analysis [29, 33] (Fig 1). The most common reasons for the exclusion of studies were study designs (e.g., reviews, commentaries, case series, or case reports) and missing data (e.g., data lacking exact serum biomarkers value description and/or lack of response to our requests by the corresponding author).

### Characteristics of the included studies

All studies included were RCTs or CCTs published in English. Duration of the follow-up period was at least 1 month, with 2 studies [31, 35] including a 3-month and a 6-month follow-up, respectively, although one study was carried out for 12 months [34]. Inclusion criteria would have included studies with other inflammatory serum biomarkers; however, none were available that met the inclusion criteria, and hence all studies included were of subjects with serum hs-CRP or CRP, IL-6 and TNF-α data. The largest studies in terms of sample size of the above data were Sun et al. [33], with 157 participants and Chen et al. [29], with 134. These two studies reported that PT can reduce serum inflammatory cytokines in T2DM individuals. Of the nine studies included, four reported CRP data, three reported hs-CRP, four reported TNF-α and four reported IL-6. Table 1 describes details of the included studies, such as number of participants, methods, definitions for periodontitis, type of periodontal interventions, outcomes, and conclusions. Only three studies described sample size calculation [29, 35, 36].

**Table 1:** Characteristics of included studies.

Study	Participants	Periodontitis definition	Methods	Interventions	Outcomes	Conclusions
**O'Connell et al., 2008. [5]**	30	At least 1 site with PD ≥ 5mm and 2 teeth with AL ≥ 6mm.	RCT, 2 groups with T2DM. 3-month follow-up.	Full-mouth SRP (15) or SRP in combination with doxycycline (15). The SRP sessions were performed by the same operator. Oral hygiene reviewed twice a month.	Significant reduction for IL-6 in both groups. No significant difference between groups.	Periodontal therapy may influence in the reduction of serum inflammatory markers.
**Dağ et al., 2009. [30]**	45	PD ≥ 5mm in at least 4 sites and AL ≥ 3mm in at least 4 sites.	CCT, 30 T2DM (15 poorly controlled and 15 well-controlled) and 15 systemically health with periodontitis. 3-month follow-up.	Oral hygiene, non-surgical periodontal therapy (SRP). The SRP sessions were performed by the same operator.	TNF-α levels significantly decreased in all groups. No significant difference between groups.	Periodontal therapy can decrease systemic inflammation through TNF-α.
**Katagiri et al., 2009. [31]**	49	At least two sites with PD ≥ 4 mm, indicating mild to severe periodontitis.	RCT, 2 groups with T2DM (only one treated); 1-, 3- and 6-month follow-up. Group 1—intervention group (32). Group 2—non-intervention group (17).	Oral hygiene, supra- and subgingival debridement; minocycline ointment during intervention sessions. No report about who performed the therapy.	No significant difference for serum hs-CRP levels between intervention and control groups at each point period (1, 3, and 6 months).	hs-CRP levels do not decrease after periodontal therapy.
**Kadesler et al., 2011. [32]**	45	At least four teeth in each jaw with a PD ≥ 5mm, AL ≥ 4mm, at least two single-rooted teeth with a PD of 6 to 9 mm, and BOP.	CCT, 25 T2DM (13 well-controlled; 12 poorly controlled) and 15 healthy. All of them with chronic periodontitis. 3-month follow-up.	Non-surgical periodontal treatment: oral hygiene instructions and SRP.	CRP levels were similar at baseline and after 3 months in well-controlled and systemically health groups, whereas higher in poorly controlled group. Serum TNF-α levels decreased slightly in all groups after 3 months without significant differences. Serum IL-6 decreased significantly after 3 months in well-controlled and systemically health groups.	Decreases in circulating pro-inflammatory molecules reflected a positive improvement in the glycemic control of poorly controlled patients with diabetes.
**Sun et al., 2011. [33]**	157	PD > 5 mm, in more than 30% of sites; AL > 4 mm, or over 60% teeth with PD > 4mm and AL > 3mm.	RCT, 2 groups. 3-month follow-up. Group T2DM-NT—not treated (75). Group T2DM-T—treated (82).	Oral hygiene, full-mouth SRP, periodontal flap surgery when indicated. Antibiotics (tinidazole + ampicillin) for 3 days before and after interventions. The interventions were performed by one periodontist.	After 3 months, the levels of hs-CRP, IL-6 and TNF-α significantly decreased (P<0.05) in T2DM-T group, when compared to T2DM-NT group.	Periodontal intervention can reduce serum inflammatory cytokines levels.
**Auyeung et al., 2012. [34]**	75	< 2 affected teeth with AL ≥ 6mm and < 1 affected tooth with PD ≥ 5mm were assigned to the group with mild periodontal disease. > 2 teeth with AL ≥ 6mm and >1 tooth with a PD ≥ 5mm were assigned to the group with moderate to severe periodontal disease.	CCT, 21 patients in the mild group and 54 in the moderate to severe periodontitis underwent non-surgical periodontal therapy. 12-month follow-up.	Oral hygiene instructions, SRP, professional plaque-control program was performed at 3, 6, 9 and 12 months post-therapy.	CRP levels were significantly different at examination times for the whole cohort	No significant positive associations between metabolic and inflammatory parameters at 12 months post-therapy were found.
**Chen et al., 2012. [29]**	134	Chronic periodontitis according to AAP, with a mean clinical AL ≥ 1 mm and at least 16 teeth.	RCTGroup 1—non-surgical periodontal therapy and additional subgingival debridement at the 3-month follow-up. Group 2—non-surgical periodontal therapy and supragingival prophylaxis at the 3-month follow-up. 1-, 3-, 5- and 6-month follow-up. Group 3—no intervention throughout the study.	Group 1—non-surgical periodontal treatment (SRP at baseline and additional subgingival debridement after 3 months. Group 2: non-surgical treatment at the initial visit and only supragingival prophylaxis after 3 months. The control group received no treatment measure or formal hygiene instructions until the end of the study.	Both treatment groups (1 and 2) had significantly lower hs-CRP after 6 months. TNF-α showed no statistically significant difference.	Non-surgical periodontal therapy can improve circulating inflammatory status.
**Koromantzos et al., 2012. [35]**	60	Moderate to severe periodontal disease (8 sites with probing depth ≥ 6mm, and four sites with AL ≥ 5mm, distributed ≥ 2 different quadrants.	RCT: IG (intervention) and CG (control group-only supragingival cleaning). 1-, 3- and 6-month follow-up.	Oral hygiene instruction, IG (non-surgical periodontal full-mouth SRP) and CG (minimal treatment group-supragingival cleaning).	After log-transformed hs-CRP values there was a reduction of 0.34 in the IG group versus 0.08 increases in the CG group. The differences between groups were not statistically significant.	Non-surgical periodontal therapy did not improve hs-CRP levels.
**Lin et al., 2012. [36]**	28	At least 20 teeth remaining in the mouth and five or more teeth with PD ≥ 5mm.	RCT: SRP (14) and SRP + subgingival minocycline gel (14). 6-month follow-up.	SRP and SRP+ subgingival minocycline administration.	Small changes in log CRP levels for both groups. IL-6 increased in SRP group and slightly decreased in SRP + minocycline group. These differences were not statistically significant.	Non-surgical periodontal therapy has no significant effect on plasma levels of IL-6 and CRP.

PD—probing depth; AL—attachment level; RCT—randomized clinical trial; CCT—controlled clinical trial; T2DM- type 2 diabetes mellitus; CRP—reactive protein; hs-CRP- high sensitive c-reactive protein; SRP scaling and root planing; IL-6—interleukin 6; TNF-α—tumor necrosis factor alpha; hs-CRP—high sensitive C-reactive protein; CRP—C-reactive protein.

### Types of outcome measures

Included studies observed hs-CRP or CRP, IL-6 and TNF-α (inflammatory serum biomarkers) as primary outcomes.

### Therapy modalities

All studies described similar non-surgical periodontal therapy based on oral hygiene instruction, supragingival cleaning and scaling and root planing (SRP). Four studies combined antimicrobial adjunction with SRP.

O’Connel et al. (2008) [5] combined SRP with systemic doxycycline, Sun et al. (2011) [33] combined it with systemic tinidazole plus ampicillin, and both Katagiri et al. (2009) [31] and Lin et al. (2012) [36] combined it with topical minocycline.

### Quality assessment of included studies

Randomization was performed in all RCTs included, but only three of them appropriately described the sequence of generation and allocation concealment [29, 35, 36]. In fact, these three studies also described adequate methods of examiner masking and were therefore considered at low risk of bias. Three of the studies included [30, 32, 34] were not described as RCT and were hence deemed as being at high risk of bias due to lack of description about randomization scheme, allocation of therapy group concealment, masking of examiners, withdrawals and missed follow-up as described by Cochrane group [27]. Follow-up period and dropouts were clearly described by all studies included.

### Effects of periodontal therapy (individual outcomes)

All treatment group interventions consisted of non-surgical periodontal therapy with or without adjunctive topical or systemic antibiotics, while only one study included surgical therapy. Eight of nine studies were parallel arm two group studies, while one study [29] included three therapy groups. Of these three group studies, group 1 received SRP at baseline and additional subgingival debridement at the 3-month follow-up, while group 2 only received prophylaxis at 3-months. The third group received no intervention throughout the study period. Of the two group studies, three included SRP only [30, 32, 34], one study described minimal treatment group as supragingival cleaning [35]. The remaining studies included adjunctive antibiotics. O’Connell et al. (2008) [5] treatment protocol used SRP in combination with doxycycline. Katagiri et al. (2009) [31] used topical minocycline during intervention sessions. Sun et al. (2011) [33] treatment included flap surgery (“when indicated”) extraction of hopeless teeth, occlusal adjustment and tinidazole plus ampicillin for 3 days before and after periodontal interventions. Table 1 shows characteristics regarding the effect of PT on serum inflammatory markers. The studies by O’Connell et al. (2008) [5], Dağ et al. (2009) [30], Kardesler et al. (2010) [34], Sun et al. (2011) [33], and Chen et al. (2012) [29] reported statistically significant reductions on IL-6 and/or TNF-α and/or CRP serum levels after non-surgical periodontal therapy. The studies by Katagiri et al. (2009) [31], Kadesler et al. (2010) [32], Auyeung et al. (2012) [34], Chen et al. (2012) [29] Lin et al. (2012) [36] and Koromantzos et al. (2012) [35] reported no statistically significant difference on serum hs-CRP /CRP and/or TNF-α after PT follow-up. Due to the marked heterogeneity between studies and different outcomes regarding inflammatory mediators, it was only possible to combine the studies into a meta-analysis for TNF-α (two RCTs) and CRP (four RCTs). With regards to IL-6 and data from CCTs, the heterogeneity was too high and studies too few to reach consistent results. O’Connell et al. (2008) [5]—the authors performed two-sample Student’s t-test or Mann-Whitney U test for comparison of means between the two groups. The paired Student’s t-test or the Wilcoxon rank-sum test was used to compare baseline values with those after 3 months. Results showed a significant decrease in IL-6 serum values after 3 months of follow-up (p = 0.005).

Dağ et al. (2009) [30]—The authors performed Wilcoxon signed-rank test for dependent variables, one-way ANOVA (Post-hoc analysis) as well as Pearson’s correlation test for independent variables. The levels of TNF-α decreased after 3 months of periodontal treatment in the three groups evaluated (T2DM poorly controlled p = 0.007, T2DM well controlled p = 0.01 and systemically healthy p = 0.001).

Katagiri et al. (2009) [31]—The authors performed Wilcoxon signed-rank test to compare the changes of all parameters from baseline to 1, 3 and 6 months. Results of this analysis showed no significant changes in hs-CRP between intervention and control groups after the follow-up period (p>0.05).

Kardesler et al. (2010) [32]—Analysis of variance, Kruskal-Wallis test and Mann-Whitney U test with Bonferroni correction were used to compare and correlate biochemical analysis. CRP exhibited higher levels for poorly controlled group (P<0.05), whereas well-controlled group and systemically healthy exhibited similar CRP values (P>0.05). Serum TNF-α decreased slightly after periodontal therapy without significant differences (P>0.05) and IL-6 serum levels showed significantly lower values after 3 months in both well-controlled and systemically healthy groups (p<0.05).

Sun et al. (2011) [33]—One way ANOVA was performed, followed by LSD multiple comparison or Student’s t-test (independent samples) to estimate statistical significance between means. All inflammatory mediators evaluated (hsCRP, TNF-α, and IL-6) decreased significantly after 3 months of periodontal intervention in treated group (T2DM-T) when compared to the group without periodontal treatment (T2DM-NT) (p<0.01).

Auyeung et al. (2012) [34]—Comparisons of inflammatory parameters between baseline and 12 months post-therapy were analyzed by paired t-test. The results of this CCT showed no difference between CRP values at baseline and 12 month post-therapy for the group with moderate-to-severe periodontitis (p = 0.62).

Chen et al. (2012) [29]—ANOVA was used to analyze immunologic and metabolic variables. Both groups (group 1—non surgical therapy + additional subgingival debridement after 3 months; group 2—non surgical therapy + supragingival prophylaxis at 3 months) showed lower hs-CRP at 6 months (p<0.05), whereas hs-CRP showed no significant difference when compared with the control group after 6 months (group 3—without intervention). The results for TNF-α showed no statistical difference in any group after 6 months (P>0.05).

Koromantzos et al. (2012) [35]—Authors performed Wilcoxon Mann-Whitney U test to analyze differences between baseline and the 6-month follow-up. With regard to log-transformed hs-CRP values at the 6-month follow-up, there was no statistical difference between baseline and groups (IG—intervention and CG—control group)(p = 0.06).

Lin et al. (2012) [36]—Authors performed latent growth curve modeling (LGCM) to test differences in changes from baseline. IL-6 values increased in SRP group and decreased in SRP + minocycline group, but there was no statistical difference after the 6-month follow-up (p = 0.17). The LGCM for log-transformed CRP showed no statistical significance for both SRP and SRP + minocycline groups after 6 months period.

### Meta-analysis

**Fig 2:**
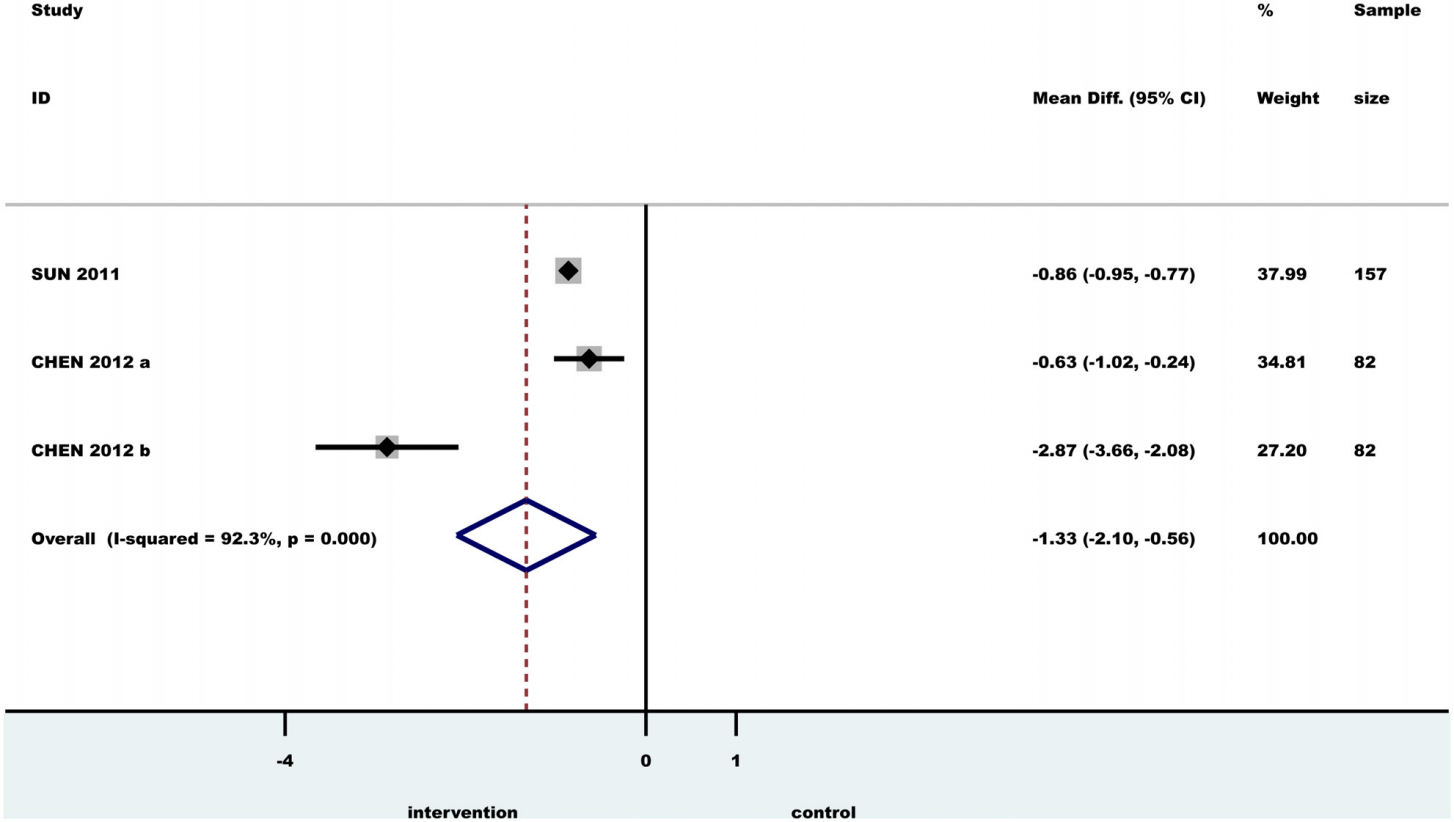
Forest plot of the difference between ΔTNF-α test and control individuals in randomized controlled trials. Horizontal lines representing 95% CI; diamond represents the overall effect size, random effects models.

**Fig 3:**
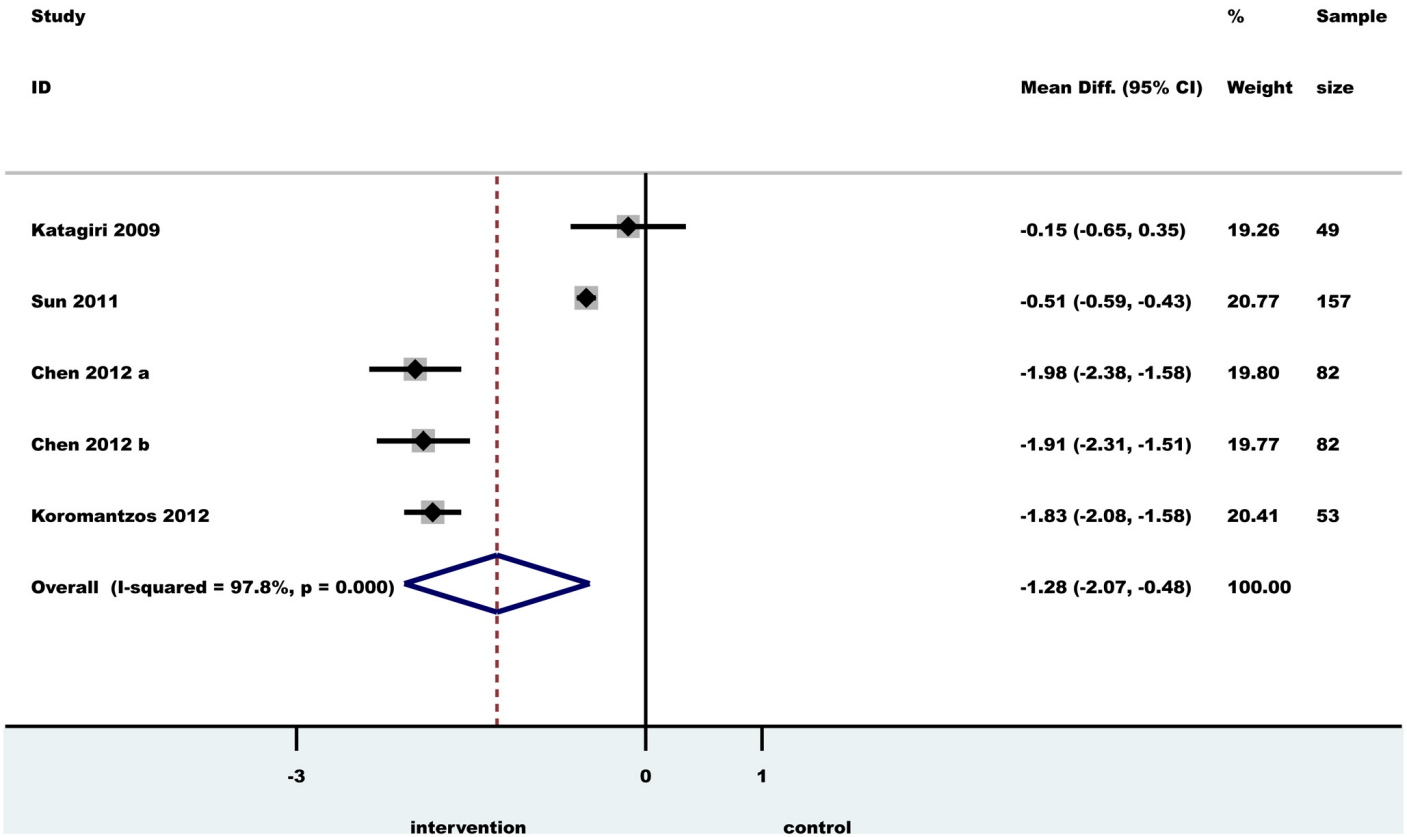
Forest plot of the difference between ΔCRP test and control individuals in randomized controlled trials. Horizontal lines representing 95% CI; diamond represents the overall effect size, random effects models.

The effect of PT on TNF-α and hs-CRP serum levels was analyzed in two meta-analysis. The mean difference (MD) and confidence interval (CI) at baseline and between PT and no periodontal treatment groups were calculated (Figs 2 and 3). A significant MD was found for both TNF-α (-1.33 pg/ml, CI: -2.10; -0.56, p<0.0001) and hs-CRP (-1.28 mg/l, CI: -2.07;- 0.48, p<0.0001). A random effects model was used to pool the data. Heterogeneity among studies was assessed using I2 statistic with 95% confidence (uncertainty) intervals [37, 38]. I2 values of 25%, 50% and 75% imply small, moderate and high heterogeneity, respectively [38]. The pooled results for meta-analysis with ‘high heterogeneity’ are not presented; instead, individual study results are presented for informal comparison. We could not assess the possible presence of publication bias with funnel plots because there were too few studies included for analysis (at least 10 studies would be necessary).

### Occurrence of adverse effects/complications associated with periodontal therapy

None of included studies reported the occurrence of adverse effects/complications.

## Discussion

### Summary of the main results

This is the first systematic review investigating the effect of PT on serum inflammatory mediators in people with T2DM. The main finding of the review was that hs-CRP and TNF-α were statistically significantly lowered by PT in people with type 2 diabetes when compared to controls. Only nine studies met the proposed inclusion criteria [29, 5, 30–36]. Six of them were RCTs and three were CCTs. Each study was assessed for a risk of bias. In all studies included in this systematic review, at least one serum inflammatory biomarker was available to evaluate the effect of PT on systemic levels of inflammation. The included studies analyzed TNF-α, hs-CRP and/or IL-6. It was possible to perform two meta-analysis with regards to TNF-α and hs-CRP. Data from CCTs and IL-6 studies were few and showed high heterogeneity to perform meta-analysis.

### Agreements and disagreements with previous studies

Relevant inflammatory biomarkers considered to play an important role in the pathogenesis of type 2 diabetes and insulin resistance were selected as primary outcomes [39]. The studies included in this review described 3 markers: TNF-α, hs-CRP and/or IL-6. Poor glycemic control and elevated levels of HbA1c in patients with type 2 diabetes have been associated with oxidative stress and high risk of cardiovascular diseases. Oxidative stress is associated with elevated serum levels of advanced glycation end products, which leads to release of serum IL-6, TNF-α and CRP [39]. A recent meta-analysis reported a cumulative improvement on HbA1c levels in periodontitis patients with diabetes [40]. A great debate aroused from this recent trial and put into question whether the periodontal treatment delivered was sufficient. No other biomarkers were measured in this study population.

Another recent meta-analysis showed that periodontal treatment improves endothelial function and reduces biomarkers of atherosclerotic disease [39, 40]. This review reported a statistically significant improvement in CRP and TNF-α serum levels after periodontal therapy in people with a number of comorbidities but not limited to type 2 diabetes. Both Teeuw et al. (2010) [23] and our study tend to suggest a consistent effect of periodontal therapy in reducing systemic inflammation in people suffering from periodontitis with or without other co-morbidities. These results could have a great impact in explaining the association of PD with increased risk of vascular events and complications in people with T2DM and it is worthy of further investigation.

### Quality of evidence and potential biases in the review process

An important reason for study exclusion was related with design (cases series reports or pilot trial) or lack of clarity about the diabetes type of participants. Such papers were thus excluded from this systematic review [41–51]. Similarly, a study conducted by Sun et al. (2010) [51] was excluded due to the fact that the same sample analyzed by Sun et al. (2011) [33] was included in this systematic review.

The RCTs conducted by Chen et al. (2012) [29], Lin et al. (2012) [36] and Koromantzos et al. (2012) [35] performed sample size calculation, but only one included a control group (with minimal supragingival intervention) for real evaluation of PT effects [35]. Lin et al. (2012) [36] compared intervention groups with or without antimicrobial, and Chen et al. (2012) [29] evaluated two intervention groups, with and without additional SRP after 3 months of follow-up. Neither the RTCs conducted by O’Connell et al. (2008) [5], Katagiri et al. (2009) [31] and Sun et al. (2011) [33] nor the CCTs conducted by Dağ et al. (2009) [30], Kardesler et al. (2010) [31] and Auyeung et al. (2012) [34] reported sample size calculation. Quality analysis for these studies revealed a high risk of bias. The most frequent criteria were reporting of sample size calculation and randomization. Absence of sample size calculation is particularly troubling because it means we cannot be sure that the study has been designed to avoid random error. Furthermore, without a sample size calculation, we cannot be sure that enough individuals were recruited to detect a difference in outcome between treatment groups if one exists (a Type 2 error). This is of special concern if the studies that have not reported a sample size calculation have found no difference in outcome between treatment groups. Randomization is a means of assigning participants to groups, such that the groups are balanced for known and unknown risk factors to minimize bias. In the absence of randomization the groups of subjects may differ in terms of severity of their periodontitis, and we cannot have a balance between known and unknown prognostic factors in the assignment of treatment.

Long-term randomized clinical trials evaluating the effects of PT on serum markers of diabetic individuals are scarce. This could be related to some important factors, such as difficulty in recruiting individuals that fulfill inclusion criteria; need to maintain the same medications during the follow-up period, and need of a controlled body mass index (BMI) for diabetic individuals. Another limitation is the unclear report about glycemic control during the studies’ follow-up. Despite the limitations of this study, we feel confident in ascribing a causal role to PT in improving serum inflammatory markers, but it is difficult to determine the magnitude of the likely benefit.

## Authors’ Conclusions

This study supports the hypothesis that PT reduces systemic inflammation in people with T2DM. Our findings emphasize the importance of periodontal health in the management of individuals with type 2 diabetes mellitus to reduce their long term risk of complications.

## Supporting Information

S1 PRISMA Checklist (XPS)
