# Supplementary figures and images for: Periodontal Therapy and Systemic Inflammation in Type 2 Diabetes Mellitus: A Meta-Analysis
Source: PLoS One. 2015 May 26;10(5):e0128344. doi: 10.1371/journal.pone.0128344 (PMC4444100; doi:10.1371/journal.pone.0128344)

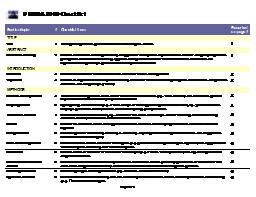

Supplement: S1 PRISMA Checklist — (XPS) [file pone.0128344.s001.xps › docProps/thumbnail.jpeg]

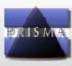

Supplement: S1 PRISMA Checklist — (XPS) [file pone.0128344.s001.xps › Resources/Images/image_0.jpg]
